# Tumours of the Bladder: Their Pathology and Diagnosis

**Published:** 1892-09-03

**Authors:** 


### SURGERY.

### TUMOURS OF THE BLADDER: THEIR PATHOLOGY AND DIAGNOSIS.

Since Sir Henry Thompson published his first case of successful "digital exploration" of the bladder in *La Semaine Médicale*, for June 18th, 1882, our knowledge of these growths has rapidly increased. Before that time a few museum specimens, and occasional reports of cases, with an account of the *post mortem* appearances, was almost the only literature on the subject. Nevertheless it has for hundreds of years been recognised in surgical writings that tumours in this organ give rise to certain recognised symptoms and to one of the most painful of deaths. It is stated, indeed, that so long ago as 1639 Covillard removed a vesical growth through a perinæal opening, and that the patient recovered.

The pathology of these tumours is far from clear. Like all organs containing mucous membrane and fibrous tissue, the bladder is subject to simple increase of these. As we find fibrous and mucous polypi in the nose and rectum, so we do in the bladder. These are found to consist of the soft gelatiniform substance seen in nasal polypi; of firmer or more vascular tissue, or of a glandular hypertrophy constituting a true adenoma. Again, the epithelial lining may be the starting point of epithelioma; scirrhus and encephaloid may occur; or the connective tissue underlying this may be possibly the seat of sarcoma.

Perhaps the most striking feature of vesical growths is their tendency to be pedunculated and fimbriated. This is not only the case with the "papillomata," or "villous" tumours, for, according to Mr. Hurry Fenwick, the more malignant kinds are often pedunculated in the early stage. This can be understood when the conditions of the new formation are realised. We will suppose that it starts from the epithelial (internal) surface, or from the tissue immediately beneath; it will therefore have on one side of it a free cavity containing a saline fluid (the urine), and on the other a constantly moving musculo-fibrous coat. The cells of the tumour would naturally multiply and spread in the direction of least resistance, and so a projection would form into the bladder cavity. The rate of increase would depend to some extent on the blood supply, and the vesical arteries send their small branches in loops or fine branches from the submucous coat inwards. An extension of this arrangement in any one spot would be apt to cause a "villous"-like growth.

It has been pointed out by Mr. Mansell Moullin that inasmuch as the bladder is developed from the original allantoic bag, it might retain the villous character of that organ in parts after its mature condition is reached, and by an overgrowth these "tufts" might result in a fimbriated tumour.

Speaking generally, we may say that the form of these growths is influenced by (1) the arrangement of the blood vessels; (2) the corrugated condition of the mucous coat (well represented by a figure in Sir Henry Thompson's "Tumours of the Bladder"); (3) the fact that increase takes place in the direction of least resistance; and (4), possibly by some persistence of a fœtal condition or a return to such.

The number of recorded cases is hardly yet sufficient for a good classification, but that of Sir Henry Thompson may be accepted as indicating our available knowledge from specimens and clinical histories. It is briefly as follows: (1) Simple mucous polypi, which are most frequently found in the young. These have the same structure as nasal polypi, but small round cells are numerous and stellate cells are rare. (2) Fimbriated papillomata, resembling the normal mucous membrane of the bladder. (3) Fibro-papillomata, more solid, and with a firmer fibrous base. (4) "Transitional," a somewhat ambiguous term for those growths which resemble the last, but have in addition a large mixture of small cells, particularly at the base. These are possibly sarcomatous. (5) Epitheliomata, which are fairly common, and are found after middle life. (6) Other forms of malignant growths.

Besides the above, perivesical tumours and hydatids sometimes occur, giving rise to cystitis and other symptoms. Guyon (*Le Bulletin Médical*, May 3rd, 1892) points out that the cysts are nearly always situated between the bladder and the rectum.

As to diagnosis, we have already said that by the imperfect means that were known 250 years ago the presence of these growths was ascertained during life, and they were even operated on. The older methods, however, could rarely be relied on for obtaining accurate knowledge, except when the condition was advanced and the symptoms correspondingly pronounced. Pain, difficult micturition, the presence of pus or blood in the urine, and no stone to account for these, were the chief signs on which to form an opinion. Examination of the rectum could sometimes detect the swelling; and the sound might, in experienced hands, give valuable information; but very few can hope to make this instrument what Sir Henry Thompson says it should be—"an elongated finger."

Digital examination through a perinæal opening, recommended by the above authority, has rendered diagnosis certain at a comparatively small risk. The urethra is opened on a grooved staff at the apex of the prostate gland, and is divided for half an inch or so. The finger is introduced through the wound, and by pressure of the other hand above the pubes the whole of the interior of the organ can be felt, and any growth which does not appear malignant may be removed.

The other method at our disposal for accurate diagnosis is Leiter's cystoscope. If this is in good working order and the urine is clear, the condition of the mucous membrane can be seen, and, with practice, understood. It seems a little doubtful, however, if this will, for some time at least, be as useful as digital exploration, for the instrument and battery are too apt to get out of gear; the field examined is a small one, the urine may be opaque, even after carefully washing out the bladder, or blood may ooze from the tumour and obstruct the view.

No doubt it is more scientific to ascertain the condition of parts without using the knife, but in these cases, whether the symptoms be due to a tumour or not, and whether removal can or cannot be effected, a perinæal opening into the bladder is usually a relief to the patient. It allows of free exit for the urine, the excretion of which may have been fraught previously with great pain; it enables the surgeon to wash out and drain the bladder, and indicates the exact condition of the interior of the organ.

